# Trace2trace—A Feasibility Study on Neural Machine Translation Applied to Human Motion Trajectories

**DOI:** 10.3390/s20123503

**Published:** 2020-06-21

**Authors:** Alessandro Crivellari, Euro Beinat

**Affiliations:** Department of Geoinformatics—Z_GIS, University of Salzburg, 5020 Salzburg, Austria; euro.beinat@sbg.ac.at

**Keywords:** seq2seq, encoder–decoder, neural networks, LSTM, trajectories, motion behavior, smart tourism

## Abstract

Neural machine translation is a prominent field in the computational linguistics domain. By leveraging the recent developments of deep learning, it gave birth to powerful algorithms for translating text from one language to another. This study aims to assess the feasibility of transferring the neural machine translation approach into a completely different context, namely human mobility and trajectory analysis. Building a conceptual parallelism between sentences (sequences of words) and motion traces (sequences of locations), we aspire to translate individual trajectories generated by a certain category of users into the corresponding mobility traces potentially generated by a different category of users. The experiment is inserted in the background of tourist mobility analysis, with the goal of translating the motion behavior of tourists belonging to a specific nationality into the motion behavior of tourists belonging to a different nationality. The model adopted is based on the seq2seq approach and consists of an encoder–decoder architecture based on long short-term memory (LSTM) neural networks and neural embeddings. The encoder turns an input location sequence into a corresponding hidden vector; the decoder reverses the process, turning the vector into an output location sequence. The proposed framework, tested on a real-world large-scale dataset, explores an effective attempt of motion transformation between different entities, arising as a potentially powerful source of mobility information disclosure, especially in the context of crowd management and smart city services.

## 1. Introduction

Trajectory recordings are gaining a continuously growing interest, allowing for a deep investigation of human mobility patterns [1,2]. The collection of motion data ranges over a variety of acquisition modalities (e.g., mobile phone traces, GPS signals, social media check-ins), often implicating the tracking of large numbers of people and, consequently, the creation of big datasets of historical motion traces. The rise of data availability has boosted the interest in human mobility [3,4], paving the way to various data mining approaches for motion behavior analysis and trajectory-related studies [5,6].

Whereas a number of different tasks have been performed on mobility traces (e.g., trajectory prediction [7,8,9,10], trajectory classification [11,12,13], motion flow modeling [14,15,16], activity recognition [17,18]), the potential uses of such data expand over a multitude of new evolving use cases. Our work is inserted in this wave of novelty and is inspired by the constantly developing field of natural language processing (NLP). The way that NLP developed its powerful methodologies and processing tools represents indeed a source of information and analytical procedures for sequence-related problems. Its sequential processing of text as a sequence of words can be generalized and adapted into different contexts as a sequential processing of generic categorical attributes. An example is represented by the introduction of neural embeddings into geographic domains and urban studies, to model locations, points of interest, functional areas and mobility traces [19,20,21,22,23].

**Figure 1:**
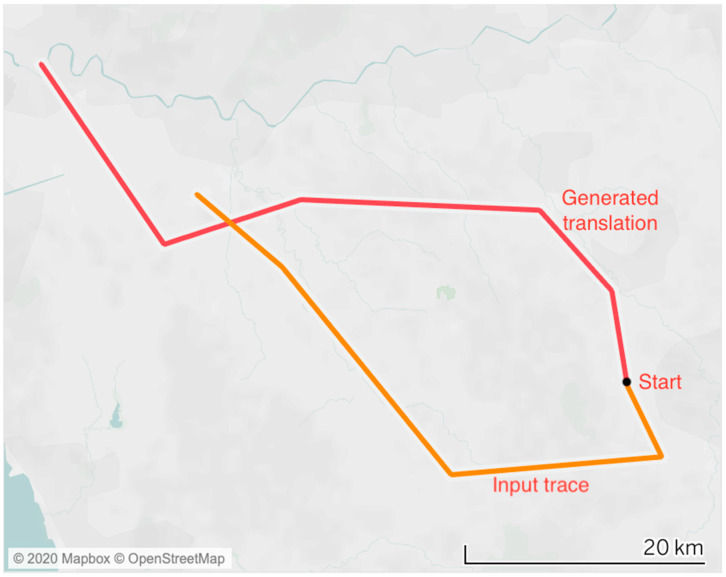
Visual representation of the trajectory “translation” task. Given a starting location and an input trace, a new output trajectory is generated from the same starting point.

Our work pushes the conceptual parallelism between NLP and trajectory analysis to a further level, introducing the concept of trajectory “translation”. As the field of neural machine translation gave birth to powerful algorithms for translating text from one language to another, we aim to assess the feasibility of transferring that approach into the context of human mobility traces. This means to adapt an NLP-inspired deep learning methodology to a completely new application, replacing the translation of text with the conversion of motion trajectories. The idea is indeed to transform individual trajectories generated by a certain category of users into the corresponding mobility traces potentially generated by a different category of users. By replacing sentences (sequences of words) with motion traces (sequences of locations), we proceed to “translate” a certain trajectory into a potentially different trajectory, or rather, generate a new trajectory given a known one, as depicted in Figure 1.

The background of tourist mobility is chosen for testing the methodology. We perform the experiment on motion traces of foreign visitors, with the goal of “translating” the motion behavior of tourists belonging to a specific nationality into the motion behavior of tourists belonging to a different nationality. The method is intended to find the representation of a function allowing to link two different motion behaviors on the same territory, and therefore to convert the first behavior into the second one. This would possibly reveal an inherent influence or dependency between the movements of tourists of different nationalities, a link between two different ways of moving over the territory. The context falls in the big picture of tourism mining, which leverages recorded space-time paths of individual tourists in order to provide valuable information on tourists’ mobility and travel behaviors. Trajectory data have been widely utilized in studies of tourists’ motion behavior [24,25,26,27,28], mainly for modeling movements between locations, as tourist destinations are involved in a complementary relationship [29,30,31]. In particular, foreign tourism is a critical area of investigation for public and private organizations, denoting significant interest as a major source of income for the tourism industry.

The proposed framework is based on the seq2seq approach, widely used in the NLP domain [32]. Specifically, the model consists of an encoder–decoder architecture based on long short-term memory (LSTM) neural networks and neural embeddings. The encoder turns an input location sequence into a corresponding hidden vector; the decoder reverses the process, turning the vector into an output location sequence. The input location sequence is intended as belonging to a chosen nationality, the output sequence refers to another chosen nationality that is different from the input one. The whole methodological procedure consists of four steps: first, raw mobility traces are pre-processed and transformed into discrete location sequences; then, the training set is defined by linking each sequence of nationality A to a sequence of nationality B on the basis of same starting locations at same time stamps; subsequently, the encoder–decoder neural network is trained through backpropagation, by feeding multiple sequences of nationality A as input to the encoder, and the corresponding linked sequences of nationality B as desired output of the decoder; finally, the model is evaluated by means of testing input sequences of nationality A, leading to automatically generating output sequences of nationality B. The experiments were conducted on a real-world dataset made of large-scale mobility traces, proving the great potentiality of such approach in the motion trajectory domain. The trace2trace methodology therefore arises as a novel beneficial trajectory-based application of deep learning tools borrowed and adapted from the NLP world.

## 2. Methodology

In this section, we proceed to present the details of our method. The problem is formally defined as follows: Given a trajectory of a certain length generated by a user of a chosen nationality starting at a specific place and time, how to predict the trajectory of the same length of a user of another nationality starting from the same place and time? As an example, if a Dutch person followed a certain time-space path, how would a Korean person move, starting from the same location at the same time?

The model is able to learn motion patterns directly from mobility traces, executing a proper trajectory conversion without any manual feature extraction or additional information. The methodological steps are illustrated in four subsections. The steps are the following:

- Trajectory pre-processing, which describes how the original traces, continuous in time and space, are transformed into discrete location sequences;
- Trajectory linking, which reports how the input and target sequences used for training the deep learning model are generated and paired;
- Seq2seq model building, which explains how to train a model that learns the relationship between input and output sequences;
- Trajectory translation inference, which focuses on the evaluation phase characterized by the generation of new output trajectories as a result of corresponding input sequences.

### 2.1. Trajectory Pre-Processing

The typical original format of a mobility trace acquired from a tracking device is characterized by a series of chronologically ordered position records, each of them defined by the time stamp of acquisition and the geographic coordinates localizing the actual position of the device. A single mobility trace can be therefore described as $T=\{p_{i}|i=1,2,3,\ldots N\}$, where $p_{i}=\left(lon_{i},lat_{i},t_{i}\right)$.

The pre-processing phase consists of a trajectory discretization process in space and time. The outcome, for each trace, is intended to be represented as a discrete location sequence, which can be subsequently handled by the neural network model. In particular, space discretization aggregates continuous values of longitude and latitude into discrete location identifiers, and time discretization transforms the continuity of time into fixed time steps. A pre-processed trajectory assumes the form of a sequence of location identifiers $\left(locID_{t},locID_{2t},locID_{3t},\ldots \right)$ referring to fixed consecutive time steps, each of them spanning a duration equal to t. In this way, time information is implicitly represented in the position along the sequence (e.g., given a time step unit resolution of 1 h, the pre-processed sequence is built by concatenating the position of the user at each consecutive hour). In general, if more than one position record were acquired within the same time step, the location associated to that specific time step is chosen as the most visited one according to the user’s position record occurrences within that time span. The choice of the time step unit length is case specific, strongly influenced by the data source type and the desired task resolution. In particular, a long unit would affect the investigation of fine resolution movements; on the other hand, the use of a short unit may substantially fragment trajectories in case of discontinuous traces, reducing the amount of data availability for training. Space resolution can also be set accordingly, possibly further discretizing it (e.g., through grid-based approaches or reference point definition) depending on the data source and the planned task configuration. This especially takes place when trajectories are sparse, and many locations comprise only very few position record occurrences. Moreover, those locations that are virtually inaccessible or irrelevant should be discarded, to avoid worthless computational effort.

The final pre-processed trajectory representation is a sequence of elements, unfolding in fixed time steps, symbolized by discrete identifiers, each of them indicating a specific unique location (or area) within a finite set of possible locations (or areas) over the territory.

### 2.2. Trajectory Linking

To properly build a training set for the neural network model, a process of trajectory linking is required. Given two nationalities A and B, the process consists of coupling trajectories of nationality A with trajectories of nationality B. The model training will be therefore performed by feeding a trajectory of nationality A as an input, and its corresponding trajectory of nationality B as a desired output. In order to construct meaningful trajectory “translations”, the criteria for linking trajectories of nationality A to trajectories of nationality B is defined on a spatial-temporal basis, namely coupling trajectory segments identified by the same starting location at the same time. This means scanning each trajectory with a sliding window and finding correspondences in terms of same locations visited at the same time by both nationalities. The sliding window, of a predetermined fixed length, is used to identify couples of subsequences whose first element refers to the same location ID at the same hour of the same day, therefore reporting that a visitor of nationality A and a visitor of nationality B were in the same place in the same day at the same hour (e.g., both visitors were in LOC_ID = 334, at 3pm on 24 March). Following this procedure, the training task consists of “translating” a subsequence referring to nationality A into a subsequence referring to nationality B, both having the same fixed length and the same first location, coinciding from a geographical and temporal point of view. In other words, given the fact that a visitor of nationality A and a visitor of nationality B are located in the same place at a certain hour, the goal is to translate the movements of nationality A in the next M hours into the movements of nationality B in the same M hours. Since the model works on a one-to-one correspondence, in case more than one subsequence identify the same starting location at the same time, trajectory linking is based on a second matching criteria relying on the most similar radius of gyration (ROG) value between subsequences. This allows selecting a trajectory couple for training whenever the same sequence can be “translated” in different ways (as also sentences can be potentially translated in similar different variants).

In practice, the data fed to the model during the training process are represented by couples of linked trajectories, one referring to nationality A and the other one referring to nationality B. Nationality A is given as an input sequence to the model, nationality B is fed as a target sequence, the desired output of the model. The training process aims to find a proper mathematical relationship that allows translating every sequence of nationality A into a corresponding sequence of nationality B. The prediction task assumes the meaning of generating a reasonable path for the unknown trajectory of nationality B, if it starts from the same location at same moment of a known trajectory of nationality A.

### 2.3. Encoder–Decoder Neural Network Model

To perform the actual transformation task, we leveraged a machine translation model [32] originally designed in NLP for translating sentences between two languages.

**Figure 2:**
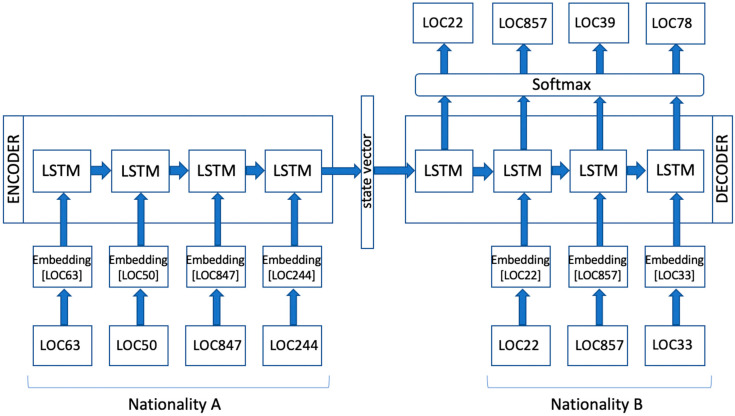
Visual exemplification of the encoder–decoder neural network model for trajectory translation.

An exemplifying visual representation is reported in Figure 2. The model consists of two blocks: an encoder and a decoder. Mobility traces of nationality A are fed to the encoder to generate the decoder output translations into nationality B. The target sequences of nationality B are instead used as inputs to the decoder, but pushed back by one step. This means that the decoder receives as an input the location that it should have output at the previous step (previous target location), regardless of which location it actually output in its prediction.

The model design makes use of the LSTM recurrent neural network [33] to encode and decode location sequences. The encoder runs an LSTM to encode inputs into a state vector; the decoder then runs another LSTM, initialized with the vector of the last encoder state, on decoder inputs. The encoder and decoder use the same LSTM cell type, but they do not share parameters.

On top of the decoder, a softmax layer is used to compute output probabilities. At each step, the decoder outputs a score for each possible location in the dataset, and the softmax layer turns these scores into probabilities (e.g., at the first step, the location LOC22 may have a probability of 40%, LOC55 may have a probability of 10%, and so on). The location with the highest probability is represented as the decoder output at that step. This reshapes the problem into a regular classification task, allowing training the model using the cross-entropy loss function. The process relies on backpropagation and mini-batch stochastic training to determine in which direction the weights are adjusted.

It is finally worth noticing that each input location, initially represented as a discrete identifier (e.g., LOC63, LOC50, LOC847, etc.), is turned into an embedding vector before being fed to the encoder or the decoder. The embedding layer indeed associates each location identifier to a specific unique low-dimensional dense vector that gets updated during the training process, just like the other model parameters in the LSTM blocks and the softmax layer.

### 2.4. Trajectory Inference

**Figure 3:**
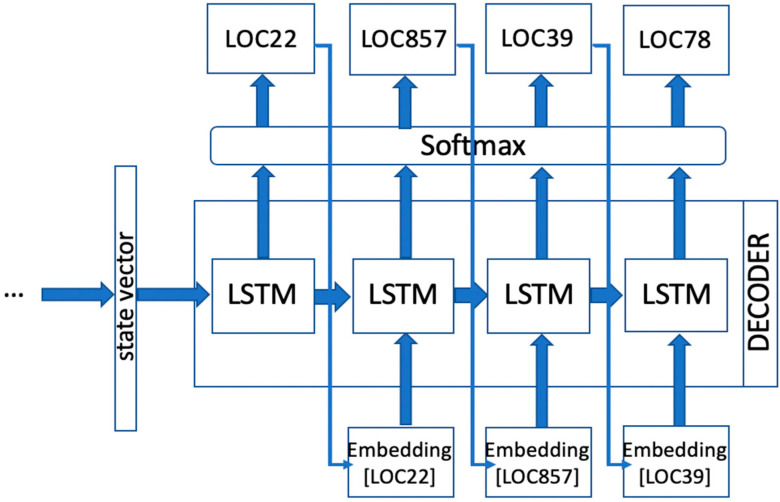
Visual exemplification of the decoder at inference time.

At inference time, no target sequence is fed to the decoder. The generation of trajectories of nationality B is solely based on the state vector deriving from encoding input sequences of nationality A. Therefore, instead of feeding the decoder with target trajectories, its input at each step is simply defined by the location that the decoder outputs at the previous step, as reported in Figure 3. The decoder input locations are always represented as embedding vectors, therefore linking the output location identifier at the previous step with the corresponding dense vector that was learned during the training process. At inference time, no model parameters are updated; the generated sequences rely on the parameter configuration that was defined at end of the training phase. The inference process therefore consists of generating a sequence of nationality B by only feeding the model with a sequence of nationality A. The generated sequence is a function of the input sequence, standing out as a plausible corresponding trajectory path that could be traveled by a visitor of a different nationality, in the same time, starting from the same location of the reference visitor.

## 3. Experiment

In the current section, we present the experiments conducted to evaluate the trace2trace approach, reporting the dataset description, the experimental settings, and the overall results. The model was implemented and executed on TensorFlow, using AWS EC2 p3.2xlarge GPU instance.

### 3.1. Dataset

We evaluated the trace2trace methodology in the context of tourist mobility analysis, leveraging motion data of visitors in a foreign country. Specifically, we made use of a real-world large-scale dataset consisting of seven months of anonymized mobile phone call detail records (CDRs) of roamers in Italy, which allowed us to track users’ geographic information through the recorded position of their device associated to any of their mobile phone activities. Each recorded event reported the coverage area of the principal antenna and the activity’s time stamp. CDR data have been widely used in research studies related to human mobility and trajectory analysis [34,35,36,37].

The sparse recorded connection events, symptoms of the typical erratic profile of mobile phone activity, posed a problem when creating location sequences during the pre-processing phase, threatening of critically fragmenting trajectories. To limit this fragmentation issue and construct proper location sequences, we set a time step unit equal to 1 h, therefore representing trajectories as sequences unfolding in 1 h time step. In case of multiple location recordings in the same hour, the current location of the user was identified as the one associated to the majority of those recordings. Considering the time step unit, the wide territory, and our interest in modeling large-scale motion activity, we also fixed a minimum spatial resolution of 2 km, projecting location points to the closest reference antenna (the one receiving the highest number of connections within the minimum spatial resolution), identifying a total of 5903 possible unique locations over the territory. Different types of data acquisition or, in general, different datasets can lead to different choices of time and space resolution, depending on their suitability for the specific application.

The construction of the final dataset version, in the form of linked trajectories, was based on coupling location sequences having the same initial location in the same hour of the same date. We set the model objective as to learn how to generate location sequences representing reasonable movements of hypothetical users in the next 6 h. The trajectory “translation” problem was indeed formulated as follows: Given a location sequence of a user of nationality A consisting of a starting location and 6 subsequent locations (representing the next 6 h), which are the corresponding 6 locations of a user of nationality B starting from the same initial location as the user of nationality A in the same hour of the same day?

Two “translation” experiments were performed: (1) translate Dutch tourists’ movements into Korean tourists’ movements, and (2) translate Dutch tourists’ movements into German tourists’ movements. Only short-term tourists were considered, located in the country for a maximum of two weeks, to avoid repetitive behaviors from the same user. The size of each dataset is based on the number of times that two users of different nationality end up in the same location at the same time. The two datasets consist of a total of 351 thousand and 913 thousand mobility traces (per nationality) respectively, representing a reasonable amount of data for realistically describing people’s traveling behaviors.

### 3.2. Experimental Settings

The encoder–decoder model was designed using two LSTM neural networks, one of them working as an encoder, and the other one as a decoder. Each LSTM was defined with a hidden size of 4000 neurons, whereas the location embedding size was set to 150 dimensions. The mini-batch training process relied on the cross-entropy cost function and Adam optimizer [38]. To evaluate the model after training, we tested its performance on previously unseen data. Each dataset was therefore split into a training set and a test set, containing 80% and 20% of the mobility traces, respectively.

In order to intuitively measure the degree of goodness achieved by the model behind the absolute results, we defined a few empirical baselines to be able to quantify how well the encoder–decoder network performed compared to the baseline approaches. The baselines were designed to highlight particular aspects of motion activity, leveraging rule-based assumptions and purely statistical models:

- Copying model. The mobility translation from nationality A to nationality B is simply performed by assuming that the traces of nationality B are the exact copy of the traces of nationality A. This baseline is useful for observing the degree of similarity between the motion behaviors of the two nationalities.
- Highest similarity model. Given a trace of nationality A in the test set, the model searches for its most similar trace (in terms of same sequentially visited locations) of nationality A in the training set and performs the translation according to its corresponding linked trace of nationality B. This baseline is useful as a measure of trace entropy, to assess the amount of repeating mobility behaviors of tourists.
- Most traveled model. Given a starting location of nationality B, its predicted trace is the most traveled path (i.e., the path that was traveled in the majority of cases by nationality B, starting from that location), regardless of any relationship with nationality A. This baseline is useful to assess the overall influence of common repeating paths performed by nationality B.
- Hidden Markov model. The locations in the trace of nationality A are treated as emissions, whereas the locations of the corresponding trace of nationality B are treated as hidden states. The problem of finding the most likely sequence of hidden states was carried out through the Viterbi algorithm. The model, based entirely on emission and transition probabilities, is traditionally widely used on sequential data, therefore representing a valuable baseline for assessing the amount of information that an encoder–decoder architecture can grasp over a purely probabilistic model.

### 3.3. Results

To have a broader view of the model performance, we reported results in the form of three accuracy score metrics. The first one represents the exact time-dependent accuracy, namely predicting the correct location at the correct time step (e.g., given a sequence of six locations referring to six consecutive hours, if four of them are predicted correctly, the accuracy of that trace will be 0.67; the overall score is the average for each testing trajectory). The second accuracy score relaxes a bit the time-dependent restrictions, namely defining a predicted location as correct if it is predicted within an interval of ±1 h with respect to the hour of the proper target location. Finally, the last score is completely time-independent, defining a predicted location as correct if it matches a target location in any of the six hours composing the sequence, regardless of its position in the trace. In other words, the first accuracy score replies to the question “how many locations are correctly predicted in the correct hour?”. The second one replies to “how many locations are correctly predicted within a ±1 h tolerance?”. The third one replies to “how many predicted locations are really visited in the next 6 h, regardless of their visiting order?”. Results are reported for both datasets: Dutch-to-Korean and Dutch-to-German.

Table 1 refers to the accuracy scores (accuracy, accuracy_±1h, accuracy_time-ind) of our encoder–decoder model and the four baselines, on the Dutch-to-Korean dataset. Our model (ENC-DEC) is shown to greatly outperform the baseline approaches, yielding a 90% improvement, compared to the best baseline result, in terms of accuracy, 86% improvement in terms of accuracy_±1h, and 60% improvement in terms of accuracy_time-ind. The most traveled model (MT) and the highest similarity model (HS) report very close accuracy score values, even slightly overcoming the hidden Markov model (HMM). The simple copying model (CP) is the worst baseline, indicating a difference in the motion behavior of the two nationalities. None of the baselines performs even close to ENC-DEC, which exceeds the best baseline’s accuracy of over 30 percentage points, demonstrating its powerful capability of translating motion patterns.

Table 2 reports, instead, the accuracy scores on the Dutch-to-German dataset. Besides a general decrease in performance, due to a higher motion behavioral variability of Germans compared to Koreans, the trend follows similar results as the previous ones, even increasing relative improvements. ENC-DEC substantially outperforms the baselines, yielding a 112% improvement, compared to the best baseline result, in terms of accuracy, 143% improvement in terms of accuracy_±1h, and 104% improvement in terms of accuracy_time-ind. In this case, baseline results are all very similar, with HS and HMM performing slightly better than the other ones. ENC-DEC still greatly overcomes the other results, once again demonstrating its ability of learning motion patterns from data, exceeding the best baseline’s accuracy of over 30 percentage points.

Moreover, we analyzed the results according to different trajectory characteristics in order to assess the performance in correspondence of different values of motion features, such as location changes and radius of gyration.

Table 3 reports the accuracy scores on the Dutch-to-Korean dataset for different numbers of location changes, namely how many times there has been a location change in the target time period. The results show a reasonable overall tendency of decreasing scores as the number of location changes increases, since the task becomes increasingly difficult for higher numbers of different locations to predict. In any case, ENC-DEC superiority is clearly visible in every category. In particular, it is worth noticing that ENC-DEC correctly predicts on average at least 3 out of 6 locations when there are 6 location changes (the user changes the current location every hour), determining an accuracy of 0.54 (the best baseline result is just 0.07), an accuracy_±1h of 0.61 (only 0.1 for the best baseline), and an accuracy_time-ind of 0.63 (only 0.13 for the best baseline), pointing out a discrete capability of modeling even highly variable traces.

Table 4 reports the corresponding results for the Dutch-to-German dataset. The overall trend is very similar to the previous one. This time, when a location change occurs at every hour, ENC-DEC correctly predicts on average 2 out 6 locations (in accordance to the general lower scores on this dataset), but still retaining a big advantage over baselines, determining an accuracy of 0.35 (only 0.05 for the best baseline result), an accuracy_±1h of 0.49 (only 0.08 for the best baseline), and an accuracy_time-ind of 0.53 (only 0.1 for the best baseline).

We also explored the performance variability with respect to the radius of gyration (ROG) of the target trajectory, expression of the area covered during traveling.

Table 5 reports the accuracies for different values of ROG, in bins of ≤3 km, 3–10 km, 10–32 km, and ≥32 km. These results reinforce the previous observations, describing a good performance of ENC-DEC even for high ROG values, particularly achieving an accuracy of 0.62 for ROG ≥32 km (only 0.19 for the best baseline), an accuracy_±1h of 0.69 (only 0.22 for the best baseline), and an accuracy_time-ind of 0.7 (only 0.25 for the best baseline). Moreover, the general big drop in performance affecting the baselines for increasing ROG values is much softer in the ENC-DEC case.

Table 6 shows the corresponding scores for the Dutch-to-German dataset. Again, ENC-DEC achieves a remarkable performance even for ROG ≥32 km, with an accuracy of 0.48 (while only 0.1 for the best baseline), an accuracy_±1h of 0.59 (only 0.12 for the best baseline), and an accuracy_time-ind of 0.6 (only 0.13 for the best baseline).

**Figure 4:**
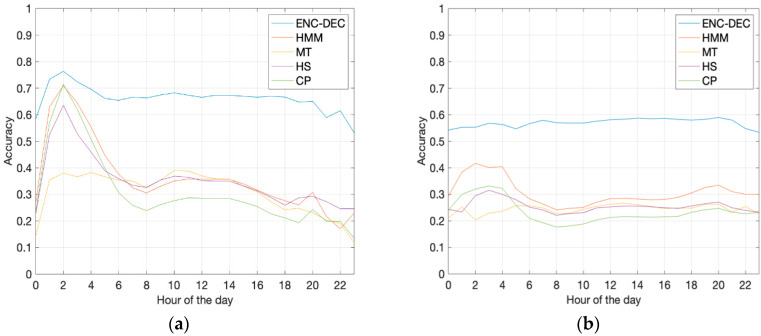
Accuracy comparison with respect to the hour of the day, on the Dutch-to-Korean dataset (**a**) and the Dutch-to-German dataset (**b**).

Finally, we observed the performance variability according to different hours of the day. Figure 4 displays the accuracy of ENC-DEC and the four baselines over time, starting from midnight, where each hour refers to the starting location prior to prediction. A similar pattern affects the baselines in both datasets, specifically reporting high accuracy values in the very early morning hours, due to the higher stationarity of mobility traces. The peak is more distinct in the Korean case (a), but it is evident even in the German case (b). On the other hand, the accuracy of ENC-DEC over time shows only minor prediction differences in the Korean case, and no peaks at all in the German case, displaying a much higher performance regularity regardless of the time of the day.

### 3.4. Discussion

We proposed a methodology for transforming individual mobility traces of a category of people into mobility traces of a different category of people by leveraging a sequence-to-sequence deep learning model adapted to process motion trajectories. The model consisted of an encoder–decoder architecture including LSTM neural network layers. We assessed such methodology on large-scale motion traces of short-term foreign tourists, demonstrating its potential feasibility on human mobility studies and applications. The experimental goal was defined as translating motion trajectories of a nationality into trajectories of a different nationality.

Travelers tend indeed to develop patterns of visit that connect multiple places-times into a sort of garland, frequently very complex. In these sequences, there are anchor places that are common to the experience of different travelers, sometimes at the same time. There are also specific clusters of places that are distinctive for some groups of people at certain times of their experience, where nationality frequently determines these differences. In this sense, the unfolding of a visit links together common and specific places in different sequences that seem to be dependent at least in part from nationalities.

By setting a few baselines modeling basic motion behavioral assumptions, we demonstrated how our method was able to greatly outperform them, revealing an actual capability of learning complex patterns along the location sequences. Specifically, predictions based on assumptions such as same movements between nationalities, repetitive movements of the target nationality, repetitive correspondences of motion activities, and common transition probabilities, were shown to perform very poorly when compared to the encoder–decoder model, explaining the presence of more complex patterns in the data, grasped by our model, besides the basic motion characteristics.

We also observed how predictability varied for different trajectory attributes. Despite the general tendency of decreasing absolute accuracy scores in correspondence of higher numbers of location changes and larger explored areas (local and stationary motion behaviors were reasonably more predictable, relying on a restricted set of possible locations), our model provided acceptable results even for very high values of ROG and location changes, always achieving a consistently much higher accuracy than the baseline approaches. In particular, the largest accuracy gap over the baselines was indeed identified exactly in correspondence of the highest values of both attributes. Moreover, while we generally observed a substantially higher score when relaxing the metric restrictions from “correct location at the correct time” to “correct location within ±1 h tolerance of the correct time”, the same behavior cannot be detected when passing from the latter to “correctly visited location regardless of time dependency”, producing only a minimal score increase. This means that the predicted locations tend to follow a specific meaningful order, where temporality is considered, and are not combined randomly to form the sequence. Finally, we studied predictability over time, where the results were split based on the hour of the day. On baselines, we noticed a general higher predictability in very early morning hours, due to the intrinsic higher degree of regularity and stationarity in the motion behaviors. However, this distinctive trend is only slightly present, or not present at all, in the case of the encoder–decoder, reporting appropriate performances over the whole day and highlighting a major improvement over baselines, especially during rush hours.

In conclusion, we assessed the feasibility of a sequence-to-sequence deep learning model for mobility trace translation, therefore introducing its use in the world of human mobility analysis. Although the purpose of the current work was mainly methodological, proposing an innovative approach into the motion analysis domain in the form of a new trajectory processing tool, our research relates to a wide variety of possible fields, ranging from location-based services, to crowd control, to destination management. A valuable implementation can be related to the generation of mobility data that are temporarily hidden or missing. Another option is strongly linked to future motion predictions, generating mobility traces of a group of people based on reliable predicted trajectories of a different group of people. These predictions can then be translated into promotions, service opportunities, demand estimations and other general needs of tourism management. In broader terms, it can improve the overall view of human collective distribution over the territory and the identification of potential crowded areas in a possible context of adjustment of supply of facilities and services.

In general, this work contributes to advance the extent of potential deep learning use in human mobility studies, disclosing encoder–decoder models as promising tools even in trajectory analysis, demonstrating their feasibility and adaptability to the motion behavioral domain.

## 4. Conclusions

Inspired by neural machine translation approaches in computational linguistics, we presented a deep learning-based methodology to mine human mobility patterns and convert motion traces of a group of users into motion traces of a different group of users. Whereas the methodology is in principle applicable to any kind of trajectory-related context in presence of two defined groups of users moving on the same territory, we leveraged the research field of tourism mobility as a case study for exhibiting the feasibility of our approach. Specifically, we processed place-based trajectories to assess the possibility of translating short-term foreign tourists’ motion activity, from a certain reference nationality into a different visitors’ nationality. The model aimed to infer the latent patterns of motion traces and find a mathematical relationship allowing transforming every sequence of nationality A into a corresponding sequence of nationality B. The process was purely data driven, the model grasped motion patterns directly from the location sequences, without any manual feature extraction. The workflow consisted of pre-processing raw traces into sequences of locations unfolding in fixed time steps, linking trajectories between the two user groups based on the rule “same geographic location at the same moment in time”, and training a seq2seq neural network composed of LSTM-based encoder and decoder layers for translating location sequences. In the context of foreign tourism analysis, our methodology was shown to greatly outperform baseline approaches based on simple motion characteristics, therefore expressing a substantial capability of grasping complex mobility patterns and modeling trace correspondences. We believe our findings can represent a starting point for further research investigations on motion behavior relationships.

Future directions can be multiple, but the primary objective will focus on solving the main limitation of our approach, that is creating output trajectories in areas characterized by the absence of training trajectories. In the current paper, a trajectory translation is possible only in correspondence of those areas where both user categories are present at the same time. Therefore, the evaluation is limited to the areas visited by both populations; it is not possible to translate movements in locations that were never seen in the training set. Finding ways to artificially generate motion traces even in absence of training trajectories is the next step to be explored, potentially utilizing deep generative models. Furthermore, a different research direction can study mobility transformation at a smaller scale, investigating the feasibility of the trace2trace model when applied to finer resolution traces in time and space (e.g., in an urban environment), potentially leveraging GPS data. Moreover, the proposed methodology can be tested for a variety of use cases dealing with motion trajectories, not limited to tourism analysis, even exploring multiple choices of technical aspects such as different trajectory-linking strategies and variable-length mobility traces. Finally, a more conceptual direction should help provide a better theoretical clarification on the inherent semantic relation of motion behaviors. A further dig into under-the-hood explanation slivers on the causes leading to the “translation choices” performed by the model may be attempted by investigating the high-dimensional space of the state vectors.

To conclude, the adaptation of neural machine translation architectures to motion translation represents a promising potential in the field of human mobility, deserving further attention and exploratory studies in the extensive domain of trajectory analysis and motion activity modeling.

## Figures and Tables

**Table 1:** Overall performance comparison on the Dutch-to-Korean dataset between our methodology (ENC-DEC) and the baseline approaches, namely copying model (CP), highest similarity model (HS), most traveled model (MT), and hidden Markov model (HMM).

Model	Accuracy	Accuracy_±1h	Accuracy_Time-Ind
CP	0.2758	0.3668	0.4538
HS	0.3470	0.4291	0.5125
MT	0.3525	0.4173	0.5125
HMM	0.3449	0.3893	0.4451
ENC-DEC	0.6710	0.8000	0.8232

**Table 2:** Overall performance comparison on the Dutch-to-German dataset between our methodology (ENC-DEC) and the baseline approaches, namely copying model (CP), highest similarity model (HS), most traveled model (MT), and hidden Markov model (HMM).

Model	Accuracy	Accuracy_±1h	Accuracy_Time-Ind
CP	0.2050	0.2928	0.3807
HS	0.2450	0.3198	0.3993
MT	0.2499	0.2921	0.3463
HMM	0.2726	0.3101	0.3725
ENC-DEC	0.5774	0.7756	0.8138

**Table 3:** Comparison of accuracy, accuracy_±1h (in round brackets), and accuracy_time-ind (in square brackets) for different numbers of location changes, on the Dutch-to-Korean dataset.

Model	1 Change	2 Changes	3 Changes	4 Changes	5 Changes	6 Changes
CP	0.4823	0.3531	0.2145	0.1455	0.0807	0.0366
	(0.6025)	(0.4749)	(0.2980)	(0.2047)	(0.1149)	(0.0638)
	[0.7151]	[0.5923]	[0.3805]	[0.2679]	[0.1550]	[0.0883]
HS	0.5913	0.4107	0.2947	0.2018	0.1270	0.0674
	(0.6798)	(0.5246)	(0.3733)	(0.2662)	(0.1715)	(0.0990)
	[0.7735]	[0.6395]	[0.4506]	[0.3329]	[0.2148]	[0.1317]
MT	0.6117	0.3942	0.3208	0.2091	0.1161	0.0567
	(0.6929)	(0.4836)	(0.3757)	(0.2586)	(0.1474)	(0.0763)
	[0.7796]	[0.6508]	[0.4531]	[0.3275]	[0.1856]	[0.1074]
HMM	0.5652	0.4545	0.2827	0.1927	0.1064	0.0300
	(0.6248)	(0.5009)	(0.3303)	(0.2289)	(0.1279)	(0.0564)
	[0.6893]	[0.5787]	[0.3830]	[0.2708]	[0.1544]	[0.0704]
ENC-DEC	0.8337	0.7037	0.6192	0.5798	0.5570	0.5358
	(0.9457)	(0.8489)	(0.7661)	(0.7109)	(0.6581)	(0.6145)
	[0.9610]	[0.8742]	[0.7924]	[0.7368]	[0.6842]	[0.6313]

**Table 4:** Comparison of accuracy, accuracy_±1h (in round brackets), and accuracy_time-ind (in square brackets) for different numbers of location changes, on the Dutch-to-German dataset.

Model	1 Change	2 Changes	3 Changes	4 Changes	5 Changes	6 Changes
CP	0.3551	0.2898	0.1780	0.1289	0.0772	0.0344
	(0.4717)	(0.4079)	(0.2661)	(0.1987)	(0.1236)	(0.0622)
	[0.5817]	[0.5209]	[0.3549]	[0.2757]	[0.1789]	[0.0901]
HS	0.4376	0.3218	0.2183	0.1576	0.1010	0.0529
	(0.5241)	(0.4263)	(0.2933)	(0.2203)	(0.1454)	(0.0776)
	[0.6102]	[0.5372]	[0.3729]	[0.2915]	[0.1959]	[0.1034]
MT	0.4643	0.3149	0.2351	0.1574	0.0942	0.0417
	(0.5189)	(0.3848)	(0.2683)	(0.1877)	(0.1140)	(0.0528)
	[0.5739]	[0.4773]	[0.3175]	[0.2286]	[0.1420]	[0.0672]
HMM	0.4441	0.3958	0.2456	0.1797	0.1025	0.0325
	(0.4985)	(0.4407)	(0.2855)	(0.2090)	(0.1211)	(0.0479)
	[0.5641]	[0.5347]	[0.3464]	[0.2620]	[0.1577]	[0.0659]
ENC-DEC	0.8014	0.6746	0.5492	0.4696	0.4022	0.3466
	(0.9307)	(0.8579)	(0.7815)	(0.7150)	(0.6320)	(0.4932)
	[0.9508]	[0.8903]	[0.8256]	[0.7655]	[0.6830]	[0.5261]

**Table 5:** Comparison of accuracy, accuracy_±1h (in round brackets), and accuracy_time-ind (in square brackets) for different values of radius of gyration, on the Dutch-to-Korean dataset.

Model	≤3 km	3–10 km	10–32 km	≥32 km
CP	0.4549	0.2151	0.1712	0.1160
	(0.6021)	(0.2972)	(0.2274)	(0.1517)
	[0.7420]	[0.3871]	[0.2837]	[0.1775]
HS	0.5167	0.2953	0.2589	0.1845
	(0.6355)	(0.3789)	(0.3237)	(0.2220)
	[0.7685]	[0.4642]	[0.3803]	[0.2477]
MT	0.5074	0.3145	0.2802	0.1930
	(0.6102)	(0.3769)	(0.3296)	(0.2125)
	[0.7846]	[0.4519]	[0.3728]	[0.2364]
HMM	0.5339	0.2940	0.2419	0.1627
	(0.5858)	(0.3359)	(0.2846)	(0.1989)
	[0.6682]	[0.3932]	[0.3269]	[0.2224]
ENC-DEC	0.7245	0.6310	0.6591	0.6250
	(0.8850)	(0.7946)	(0.7749)	(0.6927)
	[0.9180]	[0.8274]	[0.7928]	[0.6982]

**Table 6:** Comparison of accuracy, accuracy_±1h (in round brackets), and accuracy_time-ind (in square brackets) for different values of radius of gyration, on the Dutch-to-German dataset.

Model	≤3 km	3–10 km	10–32 km	≥32 km
CP	0.3783	0.1673	0.1200	0.0614
	(0.5263)	(0.2575)	(0.1660)	(0.0837)
	[0.6675]	[0.3575]	[0.2133]	[0.0983]
HS	0.4222	0.2010	0.1666	0.0980
	(0.5413)	(0.2798)	(0.2110)	(0.1195)
	[0.6742]	[0.3660]	[0.2525]	[0.1330]
MT	0.4209	0.2123	0.1799	0.0904
	(0.4970)	(0.2500)	(0.2038)	(0.1009)
	[0.6035]	[0.2952]	[0.2293]	[0.1112]
HMM	0.4609	0.2463	0.1781	0.0897
	(0.5096)	(0.2823)	(0.2118)	(0.1136)
	[0.6039]	[0.3543]	[0.2502]	[0.1270]
ENC-DEC	0.6714	0.5464	0.5588	0.4846
	(0.8819)	(0.7863)	(0.7483)	(0.5863)
	[0.9231]	[0.8384]	[0.7792]	[0.5999]
